# Evidence That Peripheral Leptin Resistance in Omental Adipose Tissue and Liver Correlates with MASLD in Humans

**DOI:** 10.3390/ijms25126420

**Published:** 2024-06-11

**Authors:** Lucia De la Cruz-Color, Jose Alfredo Dominguez-Rosales, Montserrat Maldonado-González, Bertha Ruíz-Madrigal, Martha P. Sánchez Muñoz, Vianney Alejandrina Zaragoza-Guerra, Victor H. Espinoza-Padilla, Elizabeth del C. Ruelas-Cinco, Sandra M. Ramírez-Meza, José R. Torres Baranda, María del R. González-Gutiérrez, Zamira Helena Hernandez Nazara

**Affiliations:** 1 Centro de Investigación en Biotecnología Microbiana y Alimentaria, División de Desarrollo Biotecnológico, Centro Universitario de la Ciénega, Universidad de Guadalajara, Ocotlán 47820, C.P., Mexico; lucia.delacruz@academicos.udg.mx; 2 Instituto de Investigación en Enfermedades Crónicas Degenerativas, Centro Universitario de Ciencias de la Salud, Universidad de Guadalajara, Guadalajara 44340, C.P., Mexicovhugo.epadilla@gmail.com (V.H.E.-P.); 3 Laboratorio de Investigación en Microbiología, Departamento de Microbiología y Patología, Centro Universitario de Ciencias de la Salud, Universidad de Guadalajara, Guadalajara 44340, C.P., Mexico; montserratmaldonado@yahoo.com.mx (M.M.-G.); bertha.ruiz@academicos.udg.mx (B.R.-M.); rodrigo.torresbaranda@gmail.com (J.R.T.B.); 4 Nuevo Hospital Civil de Guadalajara Dr. Juan I. Menchaca, Unidad de Cirugía Bariátrica y Metabólica, Guadalajara 44340, C.P., Mexico; cirugiapatriciasanchez@gmail.com; 5 Instituto Tecnológico y de Estudios Superiores de Monterrey, Campus Guadalajara, Escuela de Medicina y Ciencias de la Salud, Zapopan 45201, C.P., Mexico; vazg@tec.mx (V.A.Z.-G.); dra.rocio.g@tec.mx (M.d.R.G.-G.); 6 Laboratorio Estatal de Transplantes, Edificio B, Zoquipan, Zapopan 45170, C.P., Mexico; elizabethruelas029@gmail.com; 7 Coordinación de la Licenciatura en Nutrición, División de Estudios de la Salud Centro Universitario de los Valles, Universidad de Guadalajara, Ameca Km. 45.5, Ameca 46600, C.P., Mexico; sandra.ramirez4084@academicos.udg.mx

**Keywords:** leptin, leptin receptor, suppressor of cytokine signaling 3, sterol regulatory element-binding transcription factor 1, stearoyl-coa desaturase-1, patatin-like phospholipase domain-containing protein 2, metabolic dysfunction-associated steatotic liver disease, non-alcoholic fatty liver disease, metabolic dysfunction-associated steatohepatitis

## Abstract

Leptin regulates lipid metabolism, maximizing insulin sensitivity; however, peripheral leptin resistance is not fully understood, and its contribution to metabolic dysfunction-associated steatotic liver disease (MASLD) is unclear. This study evaluated the contribution of the leptin axis to MASLD in humans. Forty-three participants, mostly female (86.04%), who underwent cholecystectomy were biopsied. Of the participants, 24 were healthy controls, 8 had MASLD, and 11 had metabolic dysfunction-associated steatohepatitis (MASH). Clinical and biochemical data and the gene expression of leptin, leptin receptor (*LEPR*), suppressor of cytokine signaling 3 (*SOCS3*), sterol regulatory element-binding transcription factor 1 (*SREBF1*), stearoyl-CoA desaturase-1 (*SCD1*), and patatin-like phospholipase domain-containing protein 2 (*PNPLA2*), were determined from liver and adipose tissue. Higher serum leptin and *LEPR* levels in the omental adipose tissue (OAT) and liver with MASH were found. In the liver, *LEPR* was positively correlated with leptin expression in adipose tissue, and *SOCS3* was correlated with *SREBF1-SCD1*. In OAT, *SOCS3* was correlated with insulin resistance and transaminase enzymes (*p* < 0.05 for all. In conclusion, we evidenced the correlation between the peripheral leptin resistance axis in OAT–liver crosstalk and the complications of MASLD in humans.

## 1. Introduction

Obesity, defined as the excessive accumulation of body fat, is a modifiable risk factor for several of the leading causes of mortality worldwide [1]. It is a major health problem affecting one-third of the world’s population, and childhood obesity has increased from 4% to 18% in the last 40 years [2]. In Mexico, 7 of 10 people are overweight or obese, and this country has the highest rate of childhood obesity in the world [3].

The increase in fat stored in adipose tissue leads to the metabolic imbalance of increased adipocyte lipolysis, de novo hepatic lipogenesis, very-low-density lipoprotein cholesterol secretion, and flow of free fatty acids (FFAs) derived from the diet [4]. Hence, obesity is related to dyslipidemia, increased secretion of hormones such as leptin, and the risk of developing MASLD, formerly non-alcoholic fatty liver disease (NAFLD), defined as the ectopic accumulation of triglycerides (TG) as lipid droplets (LD) in at least 5% of hepatocytes [4,5]. The MASLD terminology will acquire strength in the future if it can infer the progression of liver fibrosis, type 2 diabetes (T2D), chronic kidney disease, and cardiovascular disease [6,7,8,9,10]. From now on, NAFLD will be referred to as MASLD, also replacing the terminology obtained from literature studies to reduce confusion.

Leptin (*LEP*) is a hormone that regulates energy balance and appetite suppression. The amount of leptin released by adipocytes is proportional to the amount of adipose tissue in the body. Leptin facilitates the coordination of enzymes involved in de novo lipogenesis, such as stearoyl-coenzyme A desaturase 1 (*SCD1*) and the transcriptional regulator sterol regulatory element-binding protein 1 (*SREBF1*), which responds to insulin and promotes TG accumulation in hepatocytes. On the other hand, patatin-like phospholipase domain-containing 2 (*PNPLA2*) catalyzes the initial step in TG hydrolysis in adipocyte and non-adipocyte LDs [11]. In this case, the function of leptin is to prevent an increase and maintain constant fat stores. Leptin activates the anorexigenic pathway through a neural network in the arcuate nucleus (ARC) of the hypothalamus [12]. Previous studies have suggested that hyperleptinemia associated with insulin resistance (IR) can facilitate the accumulation of lipids in the liver and promotes MASLD, since leptin loses its ability to cause satiety, increase energy expenditure, and decrease the body fat stored [13,14].

The failure of leptin to correct hepatic steatosis may lie in the generation of a state of resistance to this hormone in peripheral organs, possibly due to the increased expression of suppressors of cytokine signaling 3 (*SOCS3*) coupled with leptin receptor (*LEPR*) deficiency [15]. Leptin resistance has mainly been studied in animal models of the hypothalamic ARC [16]. In murine models, a lack of leptin (ob/ob mice) or its receptor (db/db mice) results in ectopic fat accumulation, leading to an increase in de novo lipogenesis and decreased mitochondrial fatty acid oxidation and progression of MASLD [17,18]. If the disease progresses, leptin can worsen the process by acting as an inflammatory and fibrogenic factor [18,19,20].

Therefore, this study evaluated the contribution of peripheral resistance and leptin axis to the progression of MASLD and/or MASH in humans.

## 2. Results

### 2.1. Characteristics of the Study Population

Table 1 shows the demographic, biochemical, and anthropometric data from the study population grouped by simple steatosis (MASLD) (n = 8), steatosis plus inflammation (MASH) (n = 11), and controls (n = 24). Clinical data revealed a predominance of women (ratio 6:1), and the average age of 36 years old; however, according to the study design, there were no significant differences in age and sex between groups. The significant differences were mainly between the MASH and control groups.

The MASH group had the highest body mass index (BMI) and waist and hip circumference measurements, and was categorized as obesity type II compared to the control and MASLD groups. Likewise, the MASH group had the highest probability of IR as determined by the homeostatic model assessment for IR (HOMA-IR) value > 2.5, insulin level of 12.0 ± 3 mUI/mL, elevated alanine transaminase (ALT) of 34.5 ± 5.3 U/L, and elevated serum leptin of 37.0 ± 9.0 ng/mL. There were no significant differences when comparing the qualitative data of abdominal adiposity and low HDL-c (*p* = 0.08 and *p* = 0.287, respectively).

### 2.2. Analysis of mRNA Expression in the Study Tissues

**Figure 1:**
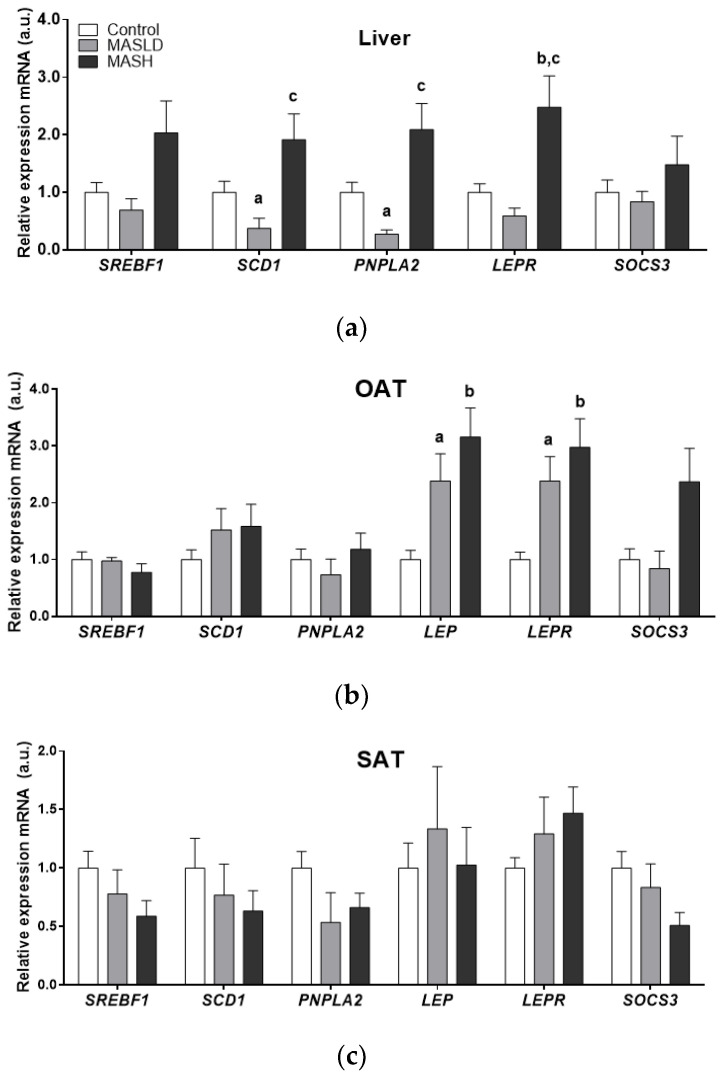
mRNA expression by study group in arbitrary units (a.u.) of liver panel (**a**), omental adipose tissue (OAT) panel (**b**), and subcutaneous adipose tissue (SAT) panel (**c**). Data from control (n = 24), MASLD (n = 8), and MASH (n = 11) groups are presented as the mean ± S.E.M. The relative expression levels were normalized to the control group. *p* < 0.05 was considered statistically significant: ^a^ Control vs. MASLD, ^b^ control vs. MASH, ^c^ MASLD vs. MASH.

The mRNA expression of *SREBF1*, *SCD1*, *PNPLA2*, leptin, *LEPR*, and *SOCS3* in the liver and adipose tissues according to the study groups is shown in Figure 1.

There was significantly higher mRNA expression of *SCD1* and *PNPLA2* in the liver samples of the MASLD and MASH groups compared to the control group. MASH biopsies also showed higher mRNA *LEPR* levels compared to the MASLD and control groups (2.5 ± 0.54-fold; *p* = 0.017). Although *SOCS3* and *SREBF1* mRNA levels showed an upward trend in MASH, these data did not reach statistical significance.

The mRNA expression of *LEP*, *LEPR*, and *SOCS3* was higher in OAT from individuals with MASH. Only *LEP* and *LEPR* genes were statistically significant (2.9± 0.5 and 3.16 ± 0.51-fold, respectively; *p* = 0.005 for both genes). These results were in accordance with the proportion of individuals with MASH and obesity type II compared to the control and MASLD groups, which fell into the overweight BMI category.

The gene expression in subcutaneous adipose tissue (SAT) did not differ significantly. However, a trend towards an increase in *LEP* and *LEPR* mRNA expression was observed between the control and MASLD groups and those individuals with MASH who had obesity type II.

At the same time, the differential expression of enzymes *PNPLA2*, *SREBF1*, and *SCD1* in adipose tissues compared to the liver was observed in the groups according to the degree of MASLD progression.

### 2.3. Protein Expression of the LEPR Long Isoform in Liver Tissue

**Figure 2:**
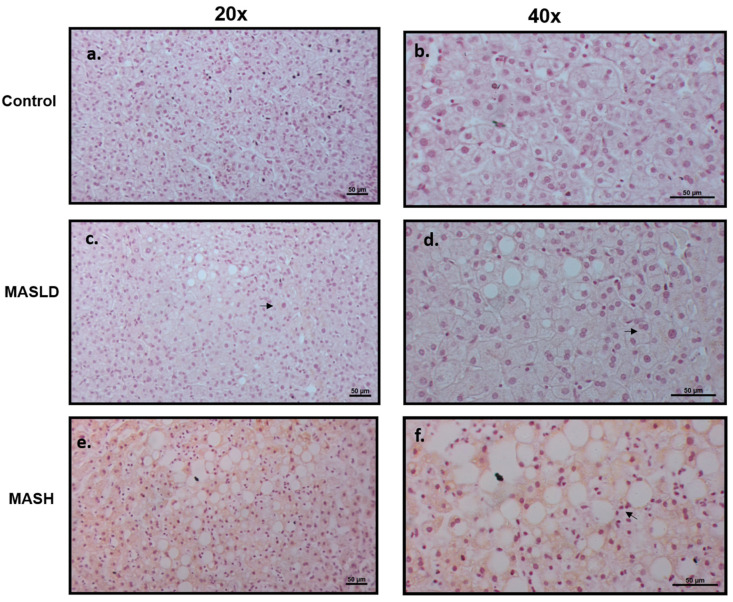
Expression of LepRb in liver tissue. Representative immunohistochemistry. Scale bars, 50 mm. (**a**,**b**) Liver biopsies of control individuals without MASLD or MASH, panel a: 20× magnification, no staining was observed; panel b: higher magnification of 40×. (**c**,**d**) MASLD nonexistent or very weak staining was observed at 20× (**c**) and 40× (**d**) magnification (see arrows). (**e**,**f**) Higher accumulation of LepRb immunostaining in liver biopsies with MASH was observed at 20× (**e**) and 40× (**f**) magnification. The immunostaining was mainly in the areas of hepatocytes with hepatic macro- and micro-lipid droplets and was negative in hepatocytes without accumulation of lipids. The arrow indicates inflammatory infiltration.

The *LEPR* long isoform (LepRb) protein expression was evaluated by immunohistochemistry in sections of liver biopsies from patients who were histologically diagnosed with or without MASLD and MASH. The LepRb antibody is specific for the epitope between amino acids 870 and 894, corresponding to the intracytoplasmic regions, so we assumed that it detects all of the transmembrane forms from LepRa to LepRd, short and long, except the soluble form, LepRe. Figure 2 shows representative photomicrographs from the control group, which did not show immunostaining of liver cells (0% of seven samples). In patients with MASLD, the staining was weak or nonexistent; positive (50% of 8 samples) staining was found in the cytoplasm of hepatocytes with LD, mainly in the zone 3 acinar, which shows a pattern of patchy immunostaining. In patients with MASH, staining was similar to MASLD (83% of six samples), with intracytoplasmic localization surrounding the lipid vacuoles, but the staining intensity was greater and the extent of immunostaining was accompanied by lobular inflammation (chi-squared and Fisher’s exact tests, *p* < 0.01). These findings confirm the role of the LepRb in the degree of severity during the progressive progression to MASH.

**Figure 3:**
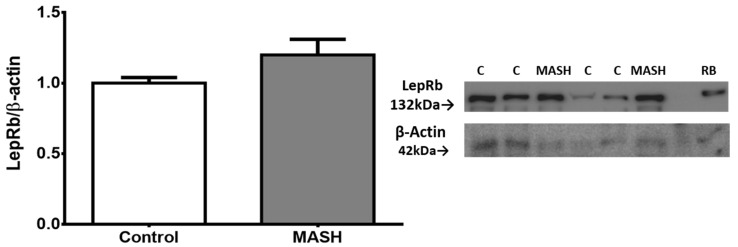
Western blot analysis showed no significant difference (Student’s *t*-test) in total protein expression of LepRb in the liver. Data from subjects with MASH (n = 5) and controls (n = 12) are presented as the mean ± SEM. The relative expression levels were normalized to the control group and b-actin for protein loading. RB indicates a sample from rat brain tissue, which served as a positive control. Representative membrane images are included.

Liver protein extracts were assessed using an antibody that recognizes the LepRb in its extracellular region between amino acids 577 and 594, which is the extracytoplasmic region. Although there was a higher level (0.19 ± 0.07 fold change) in MASH patients compared to the controls, it was not statistically significant (Figure 3).

### 2.4. Correlation between Clinical Data and mRNA Expression of Genes 

Table 2 shows that BMI had significant and positive correlations with *LEP* mRNA expression in OAT and *PNPLA2* in the liver. In addition, liver *PNPLA2* mRNA expression was positively correlated with waist circumference, fat mass, percentage fat, and serum leptin. The data in Table 2 also show the inverse expression of *SREBF1* mRNA and waist circumference between tissues. Although a positive correlation was demonstrated in liver tissue, it was negatively correlated in OAT, serving as an indirect indicator of visceral obesity.

The analysis showed that BMI was positively correlated with its covariates of weight (r = 0.928, *p* = 0.01), waist circumference (r = 0.889, *p* = 0.01), waist:hip ratio (r = 0.338, *p* = 0.05), fat mass (r = 0.919, *p* = 0.01), serum leptin (r = 0.712, *p* = 0.01), and ALT (r = 0.406, *p* = 0.05).

In the same way, while fat mass and serum leptin were positively correlated with *SCD1* mRNA expression in the liver, its expression in SAT was negatively correlated with ALT.

All of these data support the differential expression between liver and adipose tissues in crucial genes of fat deposition according to the MASLD study groups, as can be observed in Figure 1.

Likewise, leptin mRNA expression in OAT was positively correlated with the HOMA-IR and serum leptin and insulin levels.

Interestingly, the mRNA expression levels of *SOCS3* in OAT were positively correlated with HOMA-IR, as well as serum insulin, AST, and ALT levels.

At the same time, the ALT marker of liver damage in individuals with steatosis and hepatic necrosis was positively correlated with the anthropometric value of waist circumference (r = 0.381, *p* = 0.05) and other biochemical markers of liver damage, such as gamma-glutamyltransferase (GGT) (r = 0.369, *p* = 0.05), AST (r = 0.735, *p* = 0.01), and serum leptin levels (r = 0.400, *p* = 0.05).

*LEP* mRNA levels were positively correlated with the mRNA expression of *LEPR*, *SCD1*, *SOCS3*, and *PNPLA2*. *LEPR* was positively correlated with *SCD1* in OAT (Table 3). *LEPR* mRNA levels were positively correlated with the mRNA expression of *PNPLA2*, *SREBF1*, and *SCD1*. Similarly, *SCD1* expression was correlated with *PNPLA2*, *SREBF1*, and *SOCS3* in the liver. 

In particular, *LEPR* mRNA expression in the liver was positively correlated with *LEP* mRNA expression in the OAT (0.446; *p* < 0.01) and SAT (0.308; *p* < 0.05). The mRNA expression of the inhibitor *SOCS3* was positively correlated with the mRNA expression of *SCD1* (0.352; *p* < 0.05) and *SREBF1* (0.381; *p* < 0.05) in the liver. In addition, *SOCS3* mRNA expression in the liver was positively correlated with *SOCS3* mRNA in the SAT (0.352; *p* < 0.05). The increased *SOCS3* mRNA expression in the liver did not reach statistical significance.

## 3. Discussion

In this study, in a population grouped by histological progression of MASLD, we found that higher leptin mRNA levels were directly correlated with the mRNA expression of *LEPR* and the leptin inhibitor *SOCS3* in OAT. These findings were associated with metabolic alterations, mainly IR (hyperinsulinemia, HOMA-IR > 2.5), and increased serum ALT and AST levels. Moreover, the higher mRNA expression of *LEPR* in the liver was found exclusively in the MASH group, indicating hormone signaling disruption and peripheral leptin resistance in individuals with MASLD.

### 3.1. Cardiometabolic Criteria

The study population mainly consisted of women who had undergone cholecystectomy surgery and were an average of 36 years old (before the climacteric). Similarly to those in the control group, individuals with MASLD were overweight compared to MASH individuals, with grade II obesity. HDL-c levels were not significantly different among the groups, but most individuals in all groups presented hypoalphalipoproteinemia. Because of the exclusion criteria, we did not include individuals with acute liver damage. Instead, the MASH group showed elevated ALT levels, which were positively correlated with serum leptin levels. ALT has also been shown to be associated with metabolic disorders and MASLD [21,22], dovetailing with our study’s findings of the association between ALT and MASLD in perimenopausal women [23]; indeed, ALT has also been defined as predictive of metabolic alterations in slim individuals [24]. ALT was also positively correlated with serum GGT levels, visceral obesity, and mRNA expression of *SOCS3* in OAT. The individuals in the study did not meet the criteria for T2D [7]. However, the MASH group showed indications of IR and one hundred percent abdominal adiposity.

The connection between MASLD and leptin has been demonstrated in both mouse models and clinical studies. Clinical data have shown that leptin resistance associated with IR can lead to the accumulation of liver LD [13,25]. Additional studies have also demonstrated that changes in MASLD are directly linked to the interactions between insulin and leptin [26,27]. In a case–control study, high leptin levels were associated with insulin resistance and T2D progression [28]. However, some cases of steatotic liver disease (SLD) may not be related to serum leptin levels, glycemic status, or obesity [6,7]. One study did not find a link between the level of adipokine leptin and the severity of fatty liver disease [29]. Hossain et al. found that body fat was unrelated to MASLD [30]. Insulin resistance was independently associated with serum leptin levels in prediabetic males, while women in the same study showed higher leptin levels, dysfunctional beta cells, and IR [31]. The individuals in this study suffered from choledocholithiasis, which also presents with sexual dimorphism that leads to elevated leptin levels, IR, and MASLD [32]. Therefore, a limitation of this study is that the findings cannot be generalized since the sample was predominantly female. Further studies should explore gender differences. 

### 3.2. The Relationship of Leptin with MASLD and MASH

Leptin protects against MASLD by reducing lipid accumulation and increasing lipid oxidation [20,33]. Leptin prevents ectopic fat accumulation in multiple organs, not just the liver [27]. Classic studies in rodent models of dysfunctional leptin signaling and leptin-deficient ob/ob and db/db mice, which are used as mouse models of MASLD, corroborate the above findings [34,35]. Studies have shown that both the lack of leptin and its resistance lead to LD accumulation in hepatocytes, lymphocyte infiltration, and glucose metabolism disparities [36]. Leptin resistance in humans is influenced by factors such as obesity, lipodystrophy, or genetic variations, comparable to the effects seen in individuals with LEPR polymorphisms and MASLD [27,37]. While leptin has potential anti-steatotic effects, it also induces pro-inflammatory and fibrogenic responses [17,38]. This suggests that leptin may contribute to progression from simple steatosis to MASH [12,25].

Adipocytes secrete leptin in proportion to body fat accumulation, leading to higher leptin levels in individuals with obesity (hyperleptinemia). As is similar to our study, even after accounting for age and sex [39], it has been found that visceral adipose tissue leptin is linked to steatosis and its inflammatory condition or MASH [25]. We observed a gradual increase in OAT mRNA leptin levels correlated with liver damage (Figure 2b). Leptin synthesis in this tissue was positively correlated with serum insulin and leptin levels, as well as HOMA-IR (Table 2).

Leptin resistance, observed in obese individuals, leads to low-grade inflammation and steatosis [40,41]. New treatment approaches are being studied, such as reducing inflammation and steatosis with glucagon-like peptide-1 (GLP-1) inhibitors [42]. The histopathological features of liver biopsies in this study revealed that patients with MASH had areas of hepatocytes with an accumulation of intracytoplasmic macrovesicles. Additionally, there was evidence of necrosis, as indicated by ballooned hepatocytes surrounded by inflammatory infiltrates in the periportal or lobular region, particularly with lymphocyte nests. Acute cases of neutrophil infiltrate suggest an inflammatory state in these patients, wherein the beneficial antisteatotic effects of leptin are lost, leading to the expression of pro-inflammatory genes, IR, oxidative stress, and endoplasmic reticulum stress, ultimately contributing to the progression of MASH [43]. Some studies have found a correlation between increased leptin levels and factors such as age or the extent of MASLD, but not with inflammation or fibrotic severity [44]. Conversely, other studies have independently correlated the onset of fibrosis and steatosis with leptin [45]. Furthermore, additional research has shown that leptin exacerbates proinflammatory and profibrotic effects in connection to the inflammatory aspect of MASLD [46]. 

Leptin, commonly associated with diet-induced obesity, can lead to inflammation, IR, and T2D [15,47,48]. Leptin reduces the production of interleukin 10 (IL-10) and increases the production of tumor necrosis factor-alpha (TNF-α) [49]. Furthermore, myeloid cells lacking leptin signaling show improved glucose tolerance in obese mice, supporting the role of leptin as a mediator of low-grade inflammation [50]. Zhang et al. found that elevated leptin was linked to an increase in lymphocytes during the development of MASLD. This is because activated CD8+ T lymphocytes proliferate while CD4+ T lymphocytes decrease in number. Lymphocytes release granzymes that can cause reactive oxygen species in mitochondria and activate caspases, leading to pyroptosis [51]. These findings are linked to the development of MASLD and MASH. 

The research in animal models showed that inflammation in adipose and liver tissue leads to systemic inflammation [52,53]. Even after weight loss, obesity-induced immune memory persists in adipose tissue [54,55]. In animal models using high-fat diets, elevated leptin levels are linked to inflammation and MASH [56]. Leptin is not the only proinflammatory cytokine secreted by adipose tissue that initiates low-grade inflammation in obese individuals [14,56,57]. High sensitivity to leptin treatment and a high-fat diet can hinder the beneficial effects of leptin, leading to metabolic alterations like glucose intolerance [58].

### 3.3. Leptin Resistance

Leptin signaling can be both central and peripheral [59,60,61,62,63]. Mark et al. proposed the term “selective leptin resistance”, which suggests that not all leptin signaling pathways are equally affected [60]. Leptin’s sympathoexcitatory effects on the cardiovascular system remain, but its metabolic effects are limited (satiety and weight loss) [64]. Leptin also functions as a mediator of low-grade inflammation [15], while its metabolic pathway deficiencies are particularly noticeable. Leptin resistance impairs its effects, promoting obesity and inhibiting the potential effectiveness of exogenous leptin treatment [59]. Increased leptin exacerbates metabolic dysfunction, while a partial reduction in leptin improves the metabolic state [63], indicating that leptin is responsible for the inflammatory state found in obesity and subsequent metabolic changes [65].

This study and others found a strong correlation between the disruption of the leptin axis and IR in MASH. The sequence of events may be as follows: obesity hyperleptinemia, inflammation, leptin resistance, continued hyperinsulinemia postprandial to IR, and subsequent metabolic dysfunction. Higher postprandial leptin values are linked to the degree of hepatic steatosis. Observational studies have found an association between serum leptin levels and MASLD in prediabetic patients [41]. However, there is still a lack of knowledge on the progression of MASLD to MASH and the possibility of these liver diseases developing into a metabolic disorder [54,66] or remission of MASLD [67]. These points are important since MASLD is the most prevalent chronic disease worldwide [68]. This study contributes to the recognition of OAT as a source of circulating levels of the leptin axis in obesity, as a precursor event of leptin resistance, and as the leading risk factor for T2D [50]. However, since this study is cross-sectional, the aforementioned aspects should be considered cautiously.

According to the bidirectional adipoinsular axis, insulin and glucose can stimulate leptin secretion and adipocyte tissue expansion. Conversely, leptin may inhibit insulin secretion and hepatic glucose synthesis [69,70]. Leptin resistance can lead to increased insulin levels and IR, potentially resulting in T2D [71]. Insulin regulates leptin secretion, and leptin levels influence insulin sensitivity independently of weight [72]. All of these events comprise a regulatory loop. Interestingly, injecting leptin into the brain ventricles of animal models leads to minimal changes in peripheral leptin levels, but improves insulin sensitivity and blood sugar control. These data support the theory that leptin regulates glucose primarily through the central nervous system (CNS) and influences peripheral insulin sensitivity through processes independent of those affecting food intake and body weight [73].

Individuals with obesity often have hyperleptinemia and loss of leptin responsiveness at the CNS [74], which is linked to T2D, inflammation, and obesity [75]. The exact role of leptin-IR mechanisms in humans, especially in the development of metabolic diseases, is not fully understood, despite the processes shown in animal studies [76] and in the clinic [77]. Increased production of leptin is primarily regulated by high levels of insulin, while beta-adrenergic stimulation reduces leptin secretion [78]. Additionally, corticosteroids, TNF-α, and IL-1 have increased leptin secretion [79,80]. Inflammatory processes lead to resistance to both hormones. However, leptin is the one that can lead to inflammation. According to previous studies in severely obese individuals with MASLD, tissue-specific mRNA profiling has identified visceral adipose tissue-derived leptin to be involved in the development of MASH characteristics [39]. Understanding these mechanisms is crucial for preventing and treating related diseases [81,82].

Individuals with obesity have high leptin levels, but they do not respond well to external leptin treatment [59,83]. This resistance is linked to inflammation and metabolic dysfunction [84,85]. Serum leptin levels must remain within a specific range to be effective, and high leptin levels can cause unresponsiveness, even in genetically leptin-deficient mice [58]. The average rise in response usually results in a plateau when the response disappears, highlighting the importance of caloric restriction as a sensitizer [63]. The plasma leptin concentration in the cerebral spinal fluid either hits a plateau without change or does not increase when levels rise over 25 to 30 ng/mL [15,86]. Quamar et al. found that normal-weight controls had leptin levels of 10 (17.1) ng/mL, while individuals with MASLD had levels of 20.5 (21) ng/mL [87]. In this study, the control group had averages of 20.4 ± 2.5 ng/mL, and those with MASH had levels up to 37.4 ± 9 ng/mL; participants had BMI > 25 kg/m^2^ and were mostly female, and even the controls were overweight. Estrogen can increase leptin levels in rats and humans [88]; females exhibit leptin and insulin resistance rather than sensitization to leptin’s sympathoexcitatory actions [40], and threshold levels are unknown [87,89]. Hyperleptinemia itself can cause leptin resistance [14,84]. Normal leptin levels remain a topic of debate. Insulin sensitivity improves when BMI drops below 25 kg/m^2^, and fasting leptin levels decrease by 15 ng/dL; the mechanisms involve better delivery of leptin to the CNS and its access to specific neuronal groups [51,90]. As a result, a person’s usual weight and any past or present negative energy balance affect the transduction of the leptin signal [91]. Low blood levels of leptin and sensitivity as an antisteatotic effect are linked to sports [62], bariatric surgery [92], certain foods like polyphenols [74,93], neurocognitive control [94,95], long-term weight loss, and calorie restriction [96,97]. Leptin thresholds vary by person depending on genetics [90]; sexual dysmorphia [32,40]; and, recently, drugs such as GLP-1 inhibitors [42].

The body may reset its threshold for maintaining weight at a higher level of leptin due to excessive obesity [40]. Basal leptin levels are associated with BMI and reflect the body’s nutritional status. This study showed that serum leptin levels and BMI (or percentage of fat) are closely related to each other (r = 0.712, r = 0.659, *p* = 0.01, respectively). Patients with average weight but prior obesity have elevated leptin levels [89]. Even in the absence of IR or in lean people, leptin levels and their association with other adipokines, such as adiponectin, are still linked to MASLD [98]. The study also revealed that increased mRNA expression of leptin and *LEPR* in OAT and hepatic *LEPR* mRNA expression are associated with high levels of circulating leptin in people with MASH. These findings may point to peripheral and selective resistance characteristics, unlike in central leptin resistance.

### 3.4. Leptin Receptor and the SOCS3 Inhibitor

In this study, mRNA levels of *LEPR* increased as MASLD progressed to MASH in OAT and the liver, along with the expression of the leptin inhibitor *SOCS3*, but were not significant in the MASH group. Moreover, LepRb staining in hepatocytes showed patchy staining located in the cytoplasm of hepatocytes in MASLD with higher staining, with more extensive areas surrounding the lipid vacuoles in MASH. *SOCS3* mRNA levels did not increase in SAT, indicating a liver–OAT tissue axis. Tissue biopsies were taken simultaneously during the surgical procedure.

The function of *LEPR* in the central nervous system (CNS) is crucial for understanding its role in energy maintenance, metabolism, resistance, negative feedback, and immunological effects. The short isoforms (LepRa and LepRc) are responsible for leptin transport across the blood–brain barrier [99]. Leptin levels depend on the interaction and clearance mediated by the presence of those soluble receptors in an inverse correlation [13,100]. By contrast, only the long isoform (LepRb) can transduce the signal by binding to its ligand because it is the only receptor with a complete intracellular domain [101]. Leptin responsiveness could change the leptin set point by preventing leptin’s induction of its receptor, but paradoxically induces *LEPR* expression in the hypothalamic neuron targets and increases the threshold level because of negative feedback loops (*SOCS3* and the tyrosine phosphatase PTP1B) [40,85]. Central leptin resistance processes could be due to the inability of leptin to reach target cells and alter *LEPR* signaling or expression [102].

In one study, *LEPR* was reduced in peripheral blood mononuclear cells from obese compared to normal-weight individuals, suggesting the differential action of circulating leptin on these cells in dysmetabolic conditions [103]. The authors propose that decreased receptor expression is a common mechanism in metabolic dysfunction. The role of *LEPR* in peripheral organs, particularly in regulating fat storage in the liver, is crucial [19,104]. Although the expression of leptin and its receptor are positively correlated at the CNS level [105], some authors have proposed a reduction in the soluble LepR, including in individuals with MASLD [13,25].

Several studies have shown that leptin plays a crucial role in the development and progression of MASLD by interacting with LepRb [106,107], especially in MASH [6,20]. The steatotic liver processes observed in this study, accompanied by inflammation, support the observations that the *LEPR* is significantly upregulated in mouse CD4+CD8+ T cells and B cells upon activation, and leptin signaling promotes lymphocyte survival and function [108]. Leptin resistance in skeletal muscles leads to the accumulation of intramuscular TG [109,110]. An increase in *LEPR* indicates leptin resistance if it fails in its antisteatotic properties. Restoring LepRb expression in cardiac tissue improves cardiac function and reduces its TG content [111,112]. Increased *LEPR* levels are proposed as a measure against leptin resistance [113]. The upregulation of LepRb can increase sensitivity to leptin, demonstrating that glucagon receptor signaling increases LepRb. However, the beneficial effects of glucagon agonists in improving MASLD are variable [19]. Therefore, leptin resistance mediates hepatic and muscle LD presence.

This study supports the idea that increased *LEPR* indicates leptin resistance. We found higher mRNA and protein *LEPR* expression in both OAT and liver tissue. Despite an upward trend, Western blot analyses lacked significance, possibly due to antibody differences used for histopathological samples. Hence, the increased presence of *LEPR* indicates the progression to SLD and metabolic disease [114]. Despite the advances in knowledge about the *LEPR*, it is still necessary to fully understand its role in health and disease processes. Hence, previous studies agree that the loss of leptin signaling in the liver is related to metabolic disorders, such as IR, deterioration of the thyroid, and immune function [20,115]. This is also associated with increased lipoprotein lipase activity and LD accumulation in the liver. Hackl et al. demonstrated in their study that mouse models with regular expression of LepRb in the brain and low expression in the liver do not develop a steatotic phenotype; by contrast, genetic models with interrupted signaling of leptin in the brain level present elongated livers with steatosis [5]. The constant levels of leptin are a centerpiece of the brain involved in metabolism and weight control [116]. Additionally, pharmacological denervation of sympathetic pathways can prevent hepatic steatosis [117,118], and partial inhibition of *LEPR* in peripheral tissues has modest effects compared to central levels [119], highlighting the challenges in separating central and peripheral leptin resistance in a systemic process [120].

The fatty acid/Toll-like receptor-induced low-grade inflammation also inhibits STAT3 phosphorylation, the hypothalamic response to leptin, induces IR.in adipose tissue and SLD [121]. This suggests that systemic low-grade inflammation is the primary mechanism behind hormonal resistance. Additionally, leptin can cause inflammation and contribute to the M1 phenotype of macrophages [122], promoting inflammatory cell infiltration and NASH if leptin signaling is deficient [43].

After binding between leptin and LepRb in liver cells, intracellular signaling phosphorylation and activation of Janus kinase 2 (JAK2) are initiated. Three tyrosine residues (Tyr985, Tyr1077, and Tyr1138) located in the intracellular domain of LepRb are phosphorylated by JAK2. Tyr985 induces the SHP2 signaling pathway and mitogen-activated protein kinase activation, as well as recruitment of the *SOCS3* negative feedback regulator pathway; Tyr1077 mediates STAT5 activation; and Tyr1138 activates both STAT5 and STAT3 [26,123]. The immunoglobulin-like domain region is necessary for controlling body weight and metabolism, but is dispensable for immunological effects, so both functions are uncoupled [124]. Upon leptin-mediated activation of STAT3, *SOCS3* is activated, which binds to Tyr985 of LepRb, preventing the phosphorylation of JAK2 and completing the negative feedback of the pathway [125]. *SOCS3* is a protein with an SH2 domain that either directly inhibits JAK2 activity or targets the complex to the proteasome to block the signaling of specific JAK-cytokine receptor complexes [126]. An essential mediator in developing IR and leptin is *SOCS3* [127]. The same individuals in this study had elevated LepRb and *SOCS3* in the liver and OAT, in addition to metabolic dysfunction and leptin hypersecretion. 

The increase in most hormones usually triggers negative feedback. However, research has shown that leptin signaling can also initiate this process [61,128]. This study demonstrates that patients with MASH and IR have increased mRNA and protein levels of the *LEPR* in the liver, which can induce negative feedback mechanisms of cell signaling. Similar findings have been observed in the CNS of animal models, where obesity leads to hypothalamic inflammation, microglia activation, cytokine production, *LEPR* overexpression, and the induction of negative feedback signaling pathways through the action of *SOCS3* and PTP1B, as previously described [40,63,129]. The elevated levels of *LEPR* and *SOCS3* mRNA in both the OAT and liver associated with IR indicate a post-receptor mechanism with a predominance of negative feedback in peripheral tissue. Our data align with the differences between MASLD and MASH biopsies in the presence of *SOCS3* [130], as well as with studies showing that *SOCS3* inhibition prevents M1 polarization of Kupffer cells and steatotic progression to inflammation [131]. This demonstrates that comparable events occur in the liver and CNS [132,133].

The negative feedback mechanism in the CNS, particularly in the ARC, raises questions about its systemic and peripheral simultaneous occurrence [82]. *SOCS3* plays a role in liver insulin resistance and increased inflammation that accelerates simple steatosis [134]. *SOCS3* expression in adipose tissue is parallel to the degree of steatosis [135], *SOCS3*, is regulated by microRNA-650, and controls JAK/STAT3 signaling, posing a risk factor for MASLD and hepatocellular carcinoma [136]. *SOCS3* negatively regulates insulin and leptin signaling [137]. Hence, *SOCS3* is involved in the development of MASLD complications.

The positive correlations of *SOCS3* mRNA expression in OAT with AST, ALT, waist index, serum insulin levels, and HOMA-IR suggest organ crosstalk. Our results agree with the link between OAT, liver inflammation, and insulin resistance in patients with MASLD [52]. Additionally, the OAT autocrine–leptin mRNA axis is positively correlated with *LEPR* and *SOCS3* expression. However, *SOCS3* mRNA does not correlate with *LEPR* mRNA in the liver, despite elevated expression of both genes.

Interestingly, *SCD1* mRNA positively correlates with *SOCS3* and *SREBF1* in the liver. SCD1 is a crosstalk marker between the liver and adipose tissue and is a mediator of the metabolic effects of leptin-*SREBP1c-SCD1* [138]. SCD1 helps to mediate lipotoxicity with saturated fatty acids that affect the leptin response [139]. *PNPLA2* mRNA expression is a compensatory mechanism to deal with larger LD preferentially, while autophagy targets small LDs [11,140] and is positively correlated with serum leptin levels [11]. Autophagy of LD by *PNPLA2* is also a possible mediator of tissue damage [141,142]. 

## 4. Materials and Methods

### 4.1. Subjects

This cross-sectional study included 24 control subjects, 8 individuals with MASLD, and 11 with MASH, according to histopathological assessment by two expert pathologists based on the scoring system established by the Pathology Committee of the MASH Clinical Research Network [143]. Simultaneous biopsies of the liver, OAT, and SAT were obtained from each participant who underwent elective cholecystectomy surgery at the Juan I. Menchaca Civil Hospital of Guadalajara (Guadalajara, Mexico). The study protocol was approved by the ethics committee of the hospital (No. 68/HCJIM-JAL/2017) and conducted according to the latest revision of Helsinki’s declaration regarding the ethical principles for medical research in humans (Fortaleza, Brazil 2013). Exclusion criteria were the presence of acute cholecystitis or liver necrosis (determined by histopathological assessment and elevation of transaminase more than two times the upper limit of normal), cancer, endocrine including diagnosis of diabetes, infectious disease, pregnancy, alcohol intake > 20 g/day, consumption of glucose/lipid-lowering drugs, hormonal and anti-inflammatory therapies, and incomplete samples or clinical data. Individuals were selected through stratified random sampling to ensure that the age and sex ratios matched. Those individuals who met the criteria provided written informed consent before inclusion, and their clinical, anthropometric, and biochemical data were recorded. 

Biochemical data and anthropometric parameters were assessed as previously described [11]. BMI was calculated by dividing weight in kilograms by the square of the height in meters. A BMI ≥ 25 kg/m^2^ indicates overweight. Abdominal adiposity was identified by a waist circumference exceeding 80 cm for females and 94 cm for males. Low HDL-c levels, indicating hypoalphalipoproteinemia, were defined as less than 50 mg/dL for females and less than 40 mg/dL for males. Meeting at least one of these criteria is necessary for the current cardiometabolic diagnosis of MASLD or MASH [7].

Serum insulin (Monobind Inc., Lake Forest, CA, USA) and leptin levels (Quantikine^®^; R&D Systems Inc., Minneapolis, MN, USA) were determined using commercial enzyme-linked immunoassay kits according to the manufacturer’s instructions, with a coefficient of variation of < 10% for the assays and a sensitivity of 1.5 μUI/mL and 7.8 pg/mL, respectively. HOMA-IR index = (fasting insulin [μUI/mL] × fasting glucose (mg/dL) divided by 405). A HOMA-IR index cut-off ≥ 2.5 was considered an indicator of IR [144]. Cases and duplicate control samples were placed in microplates for each analysis. The iMark Microplate Reader (Bio-Rad Laboratories Inc., Hercules, CA, USA) determined the absorbance.

### 4.2. Analysis of mRNA Expression by Quantitative PCR 

Total RNA was isolated from human tissue samples using TRIzol^®^ solution (Thermo Fisher Scientific Inc., Waltham, MA, USA) and analyzed as previously described [11]. OAT and SAT biopsies were obtained from the omental region and superficial epigastric/umbilical region, respectively, while the liver sample was taken simultaneously from the left hepatic lobe of each study subject. RNA quantity and purity were estimated using the Nanophotometer P-Class (Implen GmBH, Munich, Germany). The RNA integrity was assessed using 1% agarose gel electrophoresis.

Reverse transcription assays were performed on 1 μg high-quality RNA with the Transcriptor First Strand cDNA Synthesis Kit (Roche Molecular Biochemistry, Munich, Germany), following the manufacturer’s instructions. Quantitative PCR assays were performed using the Light Cycler^®^ 96 System (Roche Diagnostics, Basel, Switzerland) with the following conditions: 10 min at 95 °C, followed by 40 cycles at two temperatures (15 s at 95 °C and 60 s at 60 °C). Cases and controls, aside from no-template samples in duplicate, were placed in microplates. The primers and probes were from Taqman^®^ Gene Expression (Applied Biosystems, Waltham, MA, USA) for the genes *LEP* (Hs00174877_m1), *LEPR* (Hs00900244_m1), *SOCS3* (Hs02330328_s1), *SCD1* (Hs01682761_m1), *PNPLA2* (Hs00386101_m1), *SREBF1* (Hs01088691_m1), and *POLR2A* (Hs00172187_m1). For *SREBF1*, the mRNA was determined for the *SREBP-1c* isoform because it predominates in the liver and adipose tissue (NCBI, UniProt). The mRNA levels were normalized to expression in the control subjects. The relative gene expression was calculated according to the 2-ΔΔCt method.

### 4.3. Liver Histology Evaluation and Human LEPR Immunohistochemistry

The liver biopsies were fixed in 4% paraformaldehyde. The specimens were processed to obtain paraffin sections for microscopic evaluation, stained with hematoxylin and eosin using standard protocols, and assessed by two expert pathologists based on the scoring of the Pathology Committee of The MASH Clinical Research Network as follows: The MASLD cut-off determination was >5% of affected hepatocytes with steatosis. The definition of MASH was lymphocyte infiltration within liver lobules of more than two in various nests, with or without ballooning hepatocytes [143].

Some paraffinized slides were treated with xylol and hydrated. The endogenous activity of peroxidase was quenched with 0.03% hydrogen peroxide in methanol solution and blocked in 5% FBS. The tissue was incubated overnight with a 1:10 dilution of anti-ObR/*LEPR* antibody (B:3) (sc-8391; Santa Cruz Biotechnology, Dallas, TX, USA), followed by anti-mouse peroxidase-labeled secondary antibody. Then, it was stained with diaminobenzidine and counterstained with hematoxylin using the ImmunoDetector DAB HRP Brown Kit (BS0003; Bio SB, Inc., Santa Barbara, CA, USA).

Twenty random fields were evaluated for quantification at 20× magnification. The immunohistochemical positive area was measured with an automated analyzer (Image Pro 6.3; Leica Qwin, Cambridge, UK). Data are expressed as percentages of the *LEPR*-stained area.

### 4.4. Protein Extraction and Western Blot Analysis

Liver protein extraction was prepared with the T-Per Tissue Protein Extraction Reagent (Cat. 78510; Pierce Chemical Co., Dallas, TX, USA) with a protease inhibitor cocktail (Thermo Fisher Scientific, Waltham, MA, USA). The protein concentration was determined using the BCA Protein Assay Kit (Cat. 23227; Thermo Fisher Scientific, Waltham, MA, USA). Protein samples (80 μg per lane) were resolved with 10% sodium dodecyl sulfate-polyacrylamide gel electrophoresis and transferred to the Immobilon-P transfer membrane (Millipore Co., Burlington, MA, USA). Membranes were incubated overnight at 4 °C with the following primary antibodies: polyclonal rabbit against LepRb (1:1000; Abcam Ltd., Cambridge, UK) and β-actin (sc-8432, 1:500; Santa Cruz Biotechnology). Peroxidase detection was performed with the Chemiluminescent HRP Substrate Kit (Cat. WBKLS0500; EMD Millipore, Burlington, MA, USA). The MicroChemi Imaging System (DNR Bio-Imaging Systems Ltd., Jerusalem, Israel) was used for imaging and digitalization. Quantitative results were obtained by densitometric analysis using ImageJ Software version 1.8.0 (National Institutes of Health, Bethesda, MD, USA). 

### 4.5. Statistical Analyses

The sample size was calculated using the formula to compare studies in order to find differences in averages, and the standard deviation was established. The number of subjects needed was five for each group according to the serum levels of leptin in patients with MASLD, as determined by Huang et al. [31], setting *p* < 0.05, a power of 80%, and a ratio of control to cases of 2:1.

Quantitative data are expressed as the mean ± standard error of the mean, and qualitative findings as frequencies and percentages. Differences between groups were analyzed using one-way analysis of variance or the chi-squared and Fisher’s exact test as appropriate, unless otherwise indicated in the footnotes of figures and tables. The post hoc Bonferroni test for ANOVA or Dunnett’s T3 test were applied after assuming variances as equal or not, as appropriate. Pearson’s coefficient assessed correlations between continuous variables. All statistical analyses were performed using SPSS 20.0 for Windows (IBM Corp., Armonk, NY, USA), and graphics were created with GraphPad Prism version 8.3.1 (GraphPad Software, San Diego, CA, USA).

## 5. Conclusions

**Figure 4:**
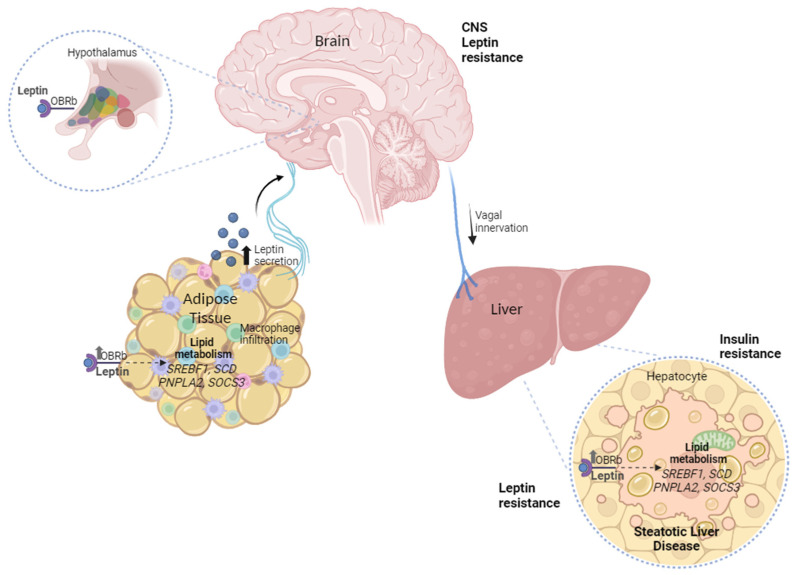
The peripheral leptin resistance model involves omental adipose tissue–liver crosstalk and the disruption of post-receptor signaling transduction in MASLD.

In conclusion, based on our findings, we propose a resistance model that involves the disruption of post-receptor signaling transduction, since we found an elevated axis of leptin hormone in serum, higher expression of its *LEPR*, and pathway inhibitor mRNA *SOCS3* levels in the OAT and liver with MASH (Figure 4). 

Hormonal resistance is characterized by the loss of metabolic regulation due to negative feedback between the hormone and target tissue. This can lead to an increase in blood levels of the hormone as a compensatory mechanism for the failure of hormonal physiological activity in the target organ [145]. Finally, the data from this study evidence the correlation between the peripheral leptin resistance axis in OAT and liver crosstalk on the complications of MASLD in humans. 

## Figures and Tables

**Table 1:** Anthropometric and biochemical analyses of the study groups.

Variable	Control n = 24	MASLD n = 8	MASH n = 11
Sex (Male/Female)	24 (3/21)	8 (0/8)	11 (3/8)
Age (years)	36.0 ± 1.7	34.3 ± 3.8	39.7 ± 1.7
Weight (kg)	69.3 ± 2.6	69.9 ± 4.1	92.2 ± 9.7
Height (cm)	161.0 ± 1.3	159.9 ± 2.4	160.6 ± 2.9
BMI (kg/m^2^)	26.7 ± 0.9	27.3 ± 1.4	35.3 ± 2.8 ^a,b^
Waist circumference (cm)	84.3 ± 3.0	86.2 ± 3.4	102.2 ± 6.1 ^a^
Abdominal Adiposity n (%)	14 (58)	6 (75)	11 (100)
Hip circumference (cm)	102.4 ± 2.5	103.8 ± 3.1	118.4 ± 6.3 ^a^
Waist:hip ratio	0.82 ± 0.02	0.83 ± 0.03	0.86 ± 0.03
Fat (%)	33.8 ± 2.2	37.7 ± 1.8	39.4 ± 3.2
Fat mass (kg)	24.6 ± 2.2	27.4 ± 3.3	33.9 ± 4.7
Glucose (mg/dL)	85.2 ± 3.3	90.4 ± 1.8	85.6 ± 3.9
Insulin (mUI/mL)	5.2 ± 0.6	8.4 ± 2.2	12.0 ± 3.0 ^a^
HOMA-IR	1.06 ± 0.14	1.90 ± 0.55	2.57 ± 0.66 ^a^
Cholesterol (mg/dL)	155.8 ± 10.0	170.3 ± 20.8	157.6 ± 17.2
Triglycerides (mg/dL)	117.0 ± 14.5	143.7 ± 20.7	118.1 ± 12.1
LDL-c (mg/dL)	89.2 ± 9.6	117.7 ± 26.4	97.3 ± 13.8
VLDL-c (mg/dL)	23.3 ± 2.9	31.3 ± 3.9	23.6 ± 2.4
HDL-c (mg/dL)	43.1 ± 3.1	38.9 ± 2.9	35.2 ± 3.4
Low HDL-c n (%)	18 (75)	8 (100)	9 (82)
ALT (U/L)	22.6 ± 2.3	21.7 ± 2.7	34.5 ± 5.3 ^a^
AST (U/L)	21.2 ± 1.9	21.0 ± 2.1	29.3 ± 5.3
GGT (U/L)	28.0 ± 5.0	37.3 ± 9.0	47.5 ± 14.6
Leptin (ng/mL)	20.4 ± 2.5	14.9 ± 3.6	37.0 ± 9.0 ^a,b^

Quantitative data are presented as the mean ± standard error of the mean (S.E.M.). Qualitative data are presented as frequencies and percentages. Sex (X = 0.24 with Fisher’s test) data are expressed as mean ± S.E.M. Abdominal adiposity: female: >80 cm, male: >94 cm; low HDL-c: female: ≤50 mg/dL, male: ≤40 mg/dL. *p* < 0.05 was considered statistically significant: ^a^ Control vs. MASH, ^b^ MASLD vs. MASH.

**Table 2:** Pearson’s correlation (R) between anthropometric and biochemical variables and the mRNA expression (a.u.) of genes in study tissues.

a.u mRNA	*PNPLA2* LIVER	*SREBF1* LIVER	*SREBF1* OAT	*SCD1* LIVER	*SCD1* SAT	*LEP* OAT	*LEPR* LIVER	*LEPR* OAT	*SOCS3* OAT
BMI (kg/m^2^)	0.376 *	ns	ns	ns	ns	0.331 *	ns	ns	ns
Waist circumference (cm)	0.337 *	0.348 *	−0.357 *	ns	ns	ns	ns	ns	ns
Waist: hip ratio	ns	0.371 *	ns	ns	ns	ns	ns	ns	0.363 *
Fat (%)	0.420 *	ns	ns	ns	ns	ns	ns	ns	ns
Fat mass (kg)	0.455 **	0.421 *	ns	0.371 *	ns	ns	ns	0.431 **	ns
Insulin (mUI/mL)	ns	ns	ns	ns	ns	0.516 **	ns	ns	0.464 **
HOMA-IR	ns	ns	ns	ns	ns	0.530 *	ns	ns	0.409 *
AST (U/L)	ns	ns	ns	ns	−0.383 *	ns	ns	ns	0.481 **
ALT (U/L)	ns	ns	ns	ns	−0.332 *	ns	ns	ns	0.513 **
Leptin (ng/mL)	0.667 *	ns	ns	0.477 **	ns	0.350 *	0.430*	ns	ns

Abbreviation: arbitrary units (a.u.) of mRNA expression. Significant differences * *p* < 0.05, ** *p* < 0.01. Non-significant ns.

**Table 3:** Pearson’s correlation (R) between the mRNA expression (a.u.) of genes in study tissues.

*OAT*	*LEP* OAT	*LEPR* OAT	*LEPR* LIVER	*SCD1* LIVER
*LEPR*	0.692 *	ns	ns	ns
*PNPLA2*	0.351 *	ns	ns	ns
*SCD1*	0.488 **	0.417 **	ns	ns
SOCS3	0.388 *	ns	ns	ns
LIVER				
*PNPLA2*	ns	ns	0.419 **	0.446 **
SCD1	ns	ns	0.577 **	ns
*SREBF1*	ns	ns	0.349 *	0.654 *
*SOCS3*	ns	ns	ns	0.352 *

mRNA expression of arbitrary units (a.u.). Significant differences * *p* < 0.05, ** *p* < 0.01. Non-significant: ns.

## Data Availability

The database used in the study is not accessible due to patient-level data protection and confidentiality and because it is still being used for clinical studies.
